# Association of Initial and Longitudinal Changes in C-reactive Protein With the Risk of Cardiovascular Disease, Cancer, and Mortality

**DOI:** 10.1016/j.mayocp.2022.10.013

**Published:** 2023-04

**Authors:** Navin Suthahar, Dongyu Wang, Joseph Pierre Aboumsallem, Canxia Shi, Sanne de Wit, Elizabeth E. Liu, Emily S. Lau, Stephan J.L. Bakker, Ron.T. Gansevoort, Bert van der Vegt, Manol Jovani, Bernard E. Kreger, Greta Lee Splansky, Emelia J. Benjamin, Ramachandran S. Vasan, Martin G. Larson, Daniel Levy, Jennifer E. Ho, Rudolf A. de Boer

**Affiliations:** Department of Cardiology, University Medical Center Groningen, University of Groningen, Groningen, The Netherlands; Department of Cardiology, Erasmus MC, University Medical Center Rotterdam, Rotterdam, The Netherlands; Cardiovascular Institute and Division of Cardiology, Department of Medicine, Beth Israel Deaconess Medical Center, Boston, MA, USA; Department of Biostatistics, Boston University, Boston, MA, USA; Department of Cardiology, University Medical Center Groningen, University of Groningen, Groningen, The Netherlands; Department of Cardiology, University Medical Center Groningen, University of Groningen, Groningen, The Netherlands; Department of Cardiology, University Medical Center Groningen, University of Groningen, Groningen, The Netherlands; Cardiovascular Research Center, Massachusetts General Hospital, Harvard Medical School, Boston, MA, USA; Cardiovascular Research Center, Massachusetts General Hospital, Harvard Medical School, Boston, MA, USA; Division of Nephrology, Department of Internal Medicine, University Medical Center Groningen, University of Groningen, Groningen, The Netherlands; Division of Nephrology, Department of Internal Medicine, University Medical Center Groningen, University of Groningen, Groningen, The Netherlands; Department of Pathology, University Medical Center Groningen, University of Groningen, Groningen, The Netherlands; Division of Gastroenterology, Maimonides Medical Center, Brooklyn, NY, USA; School of Public Health, Boston University, Boston, MA, USA; Department of Medicine, Chobanian and Avedesian School of Medicine, Boston University, Boston, MA, USA; The Framingham Heart Study, Framingham, MA, USA; The Framingham Heart Study, Framingham, MA, USA; Department of Epidemiology, Boston University, Boston, MA, USA; School of Public Health, Boston University, Boston, MA, USA; Department of Medicine, Chobanian and Avedesian School of Medicine, Boston University, Boston, MA, USA; The Framingham Heart Study, Framingham, MA, USA; Department of Epidemiology, Boston University, Boston, MA, USA; School of Public Health, Boston University, Boston, MA, USA; Department of Medicine, Chobanian and Avedesian School of Medicine, Boston University, Boston, MA, USA; The Framingham Heart Study, Framingham, MA, USA; Department of Biostatistics, Boston University, Boston, MA, USA; The Framingham Heart Study, Framingham, MA, USA; The Framingham Heart Study, Framingham, MA, USA; Population Sciences Branch, Division of Intramural Research, National Heart, Lung, and Blood Institute, National Institutes of Health, Bethesda, MD, USA; Cardiovascular Institute and Division of Cardiology, Department of Medicine, Beth Israel Deaconess Medical Center, Boston, MA, USA; Department of Cardiology, University Medical Center Groningen, University of Groningen, Groningen, The Netherlands; Department of Cardiology, Erasmus MC, University Medical Center Rotterdam, Rotterdam, The Netherlands

## Abstract

**Objective:**

To evaluate the value of serial C-reactive protein (CRP) measurements in predicting the risk of cardiovascular disease (CVD), cancer, and mortality.

**Methods:**

The analysis was performed using data from two prospective, population-based observational cohorts: the Prevention of Renal and Vascular End-Stage Disease (PREVEND) study and the Framingham Heart Study (FHS). A total of 9253 participants had CRP measurements available at two examinations (PREVEND: 1997-1998 and 2001-2002; FHS Offspring cohort: 1995-1998 and 1998-2001). All CRP measurements were natural log-transformed before analyses. Cardiovascular disease included fatal and nonfatal cardiovascular, cerebrovascular and peripheral vascular events, and heart failure. Cancer included all malignancies except nonmelanoma skin cancers.

**Results:**

The mean age of the study population at baseline was 52.4±12.1 years and 51.2% (n=4733) were women. Advanced age, female sex, smoking, body mass index, and total cholesterol were associated with greater increases in CRP levels over time (*P*_all_<.001 in the multivariable model). Baseline CRP, as well as increase in CRP over time (ΔCRP), were associated with incident CVD (hazard ratio [HR]: 1.29 per 1-SD increase; 95% confidence interval [CI]: 1.29 to 1.47, and HR per 1-SD increase: 1.19; 95% CI: 1.09 to 1.29 respectively). Similar findings were observed for incident cancer (baseline CRP, HR: 1.17; 95% CI: 1.09 to 1.26; ΔCRP, HR: 1.08; 95% CI: 1.01 to 1.15) and mortality (baseline CRP, HR: 1.29; 95% CI: 1.21 to 1.37; ΔCRP, HR: 1.10; 95% CI: 1.05 to 1.16).

**Conclusion:**

Initial as well as subsequent increases in CRP levels predict future CVD, cancer, and mortality in the general population.

Cardiovascular disease (CVD) and cancer are the two major causes of death worldwide,^1^ and there is increasing recognition that several cardiovascular risk factors are also associated with an increased risk of cancer.^2-4^ Studies addressing the “common soil” hypothesis indicate that besides aging and male sex, lifestyle-related factors such as cigarette smoking and obesity are associated with a higher risk of developing both CVD and cancer.^5-7^ However, even after accounting for traditional risk factors, the residual risk for developing these disorders remains high.^8-11^

Identifying pathophysiological mechanisms common to CVD and cancer using biomarkers could facilitate the development of therapeutic interventions targeting these mechanisms,^12^ which may help reduce the residual risk of both these disorders. For instance, C-reactive protein (CRP), a marker of inflammation, is known to be involved in the pathophysiology of both CVD and cancer.^13,14^ Recently, the Canakinumab Antiinflammatory Thrombosis Outcome Study (CANTOS) trial provided evidence that anti-inflammatory therapy targeting interleukin (IL)-1b (of the CRP/IL-6/IL-1 axis) in individuals with pre-existing CVD not only lowered the risk of developing recurrent cardiovascular events^15^ but also reduced the risk of developing cancer events.^16^

Plasma CRP levels have also shown to be useful in predicting future cardiovascular and cancer risk in the general population. However, most previous studies examining associations of CRP levels with incident CVD and cancer were based on one-time measurement.^17-20^ A drawback to this approach is that a single CRP measurement only provides “snapshot information” on the systemic inflammatory burden, which may be insufficient for adequate risk characterization. Serial CRP measurements might provide additional information in this setting, but there are only a limited number of studies examining the value of longitudinal CRP changes in estimating future cardiovascular^21,22^ or cancer risk.^23^

In the current study, we leveraged data from two well-characterized, community-based cohorts with longitudinal follow-up for cardiovascular and cancer endpoints. Our objectives were to identify clinical correlates of longitudinal changes in CRP in the general population and to examine the relationship between changes in CRP levels over time with incident CVD, incident cancer, and all-cause mortality. We hypothesized that longitudinal changes in CRP levels would more accurately reflect the progression (or regression) of disease processes and would therefore provide additional information beyond a single measurement.

## METHODS

### Study Sample

The Prevention of Renal and Vascular End-Stage Disease (PREVEND) and Framingham Heart Study (FHS) are prospective observational community-based cohort studies. Individuals enrolled in PREVEND and FHS are closely monitored for the development of CVD and cancer. Specific details on study participants have been previously described.^24-26^ For the current study, we included PREVEND participants attending examination 1 (1997-1998) and examination 2 (2002-2003), and FHS participants (from the Offspring cohort) attending an earlier examination (1995-1998) and a later examination (1998-2001). Individual-level data from both cohorts were then harmonized and pooled.

From the initial sample (N=11,856), we excluded individuals with missing CRP in either of the two visits (n=2337) as well as individuals with missing clinical covariates (n=266) resulting in a sample of 9253 participants. For analyses of incident CVD, we excluded subjects with CVD before the second time-point (n=737), resulting in a final sample of 8516 participants. For analyses of incident cancer, we excluded subjects with (any type of) cancer before the second time-point (n=544), resulting in a final sample of 8709 participants.

The current study conformed to the principles drafted in the Helsinki declaration. Institutional review board approval was obtained separately for PREVEND (University of Groningen) and FHS (Boston University), and all participants provided written informed consent.

### Clinical Assessment at Baseline

Study participants underwent a comprehensive medical history, physical examination, and phlebotomy. Smoking was defined as current smoking or smoking cessation within the previous year. Body mass index (BMI) was calculated as weight/height^2^ (kg/m^2^). Blood pressure (BP) was calculated as the average of two seated measurements. Hypertension was defined as systolic BP of 140 mm Hg or higher, diastolic BP of 90 mm Hg or higher, or antihypertensive medication usage. Diabetes was defined as a fasting glucose 126 mg/dL (7.0 mmol/L) or higher, a nonfasting glucose of 200 mg/dL (11.1 mmol/L) or higher, or hypoglycemic medication usage.

### CRP Measurements

Fasting samples were obtained at each examination and stored at −80°C until testing. Plasma CRP levels were measured with a high-sensitivity assay for PREVEND participants (Dade Behring BNII nephelometer, Marburg, Germany) with a threshold of 0.175 mg/L and intra- and inter-assay coefficients of less than 4.4% and 5.7%, respectively.^27^ Serum CRP was measured with a high-sensitivity assay for the Framingham Offspring participants (Dade Behring BN100 nephelometer, Deerfield, IL, USA). A minimal detectable limit of 0.16 mg/L was set as the threshold, with intra- and inter-assay coefficients at 3.2% and 5.3%, respectively, for quality control purposes.^28^

### Ascertainment of CVD and Cancer Outcomes

Participants were followed for the development of CVD, cancer, and/or death occurring after the second timepoint.

Cardiovascular outcomes were documented using established protocols by PREVEND and FHS study investigators after review of all available hospital records.^29,30^ Incident CVD included myocardial infarction, coronary heart disease, heart failure, stroke (ischemic and hemorrhagic), and peripheral arterial disease.^29,30^ The follow-up was censored at 9 years to assure similar duration across cohorts.

Cancer cases in the PREVEND cohort were identified through linkage between PREVEND and the Dutch nationwide network and registry of histopathology and cytopathology in the Netherlands (Pathological Anatomical National Automated Archive Foundation).^31^ Cancer outcomes in the FHS were identified through surveillance of routine examinations, health updates, hospital admissions, or from death records. All available medical records and pathology reports were reviewed and coded based on topology and morphology, and were graded by two independent physicians, with discrepancies resolved after discussion and re-review of cases with a third physician.^5,32^ Incident cancer included all malignancies, except nonmelanoma skin cancers. The follow-up was censored at 15 years to assure similar duration across cohorts.

### Statistical Analysis

In the baseline table, continuous variables were presented as means ± SD or medians (25th to 75th percentiles), and categorical variables as counts (percentages). For subsequent analyses, CRP concentrations were log_e_-transformed (ln) to address right-skewed distributions. Longitudinal change in CRP was calculated as the difference of ln CRP concentrations between the two consecutive exams (ie, ΔCRP equals ln-transformed CRP_2_ minus ln-transformed CRP_1_). For further analyses, CRP concentrations as well as ΔCRP were standardized.

To examine associations between baseline parameters and ΔCRP, we used linear regression models. The initial model was adjusted for age, sex, and CRP levels at baseline. To identify clinical correlates of ΔCRP, we used a stepwise linear regression model, with the inclusion of baseline clinical covariates at *P* value less than .10 in the initial model. Age, sex, and baseline CRP levels were forced in the selection model with a retention *P* value equal to .05.

We calculated incidence rates (IRs) of CVD, cancer, and all-cause mortality across categories of longitudinal changes in CRP by dichotomizing CRP at 2 mg/L (ie, CRP less than 2 mg/L and CRP greater than or equal to 2 mg/L).^15,33^ Individuals were categorized into four groups: (1) persistently low levels (low-low), (2) changing from low to high levels (low-high), (3) changing from high to low levels (high-low), and (4) persistently high levels (high-high).

Next, we examined the association of baseline CRP and ΔCRP with incident outcomes using Cox proportional hazards models. Baseline CRP and ΔCRP were entered simultaneously in all models. A cohort identifier variable was also added to all regression models to account for batch effects. Model 1 adjusted for age (at baseline) and sex. Model 2 additionally adjusted for the following baseline covariates: smoking, BMI, total cholesterol, lipid-lowering medication, systolic BP, antihypertensive medication, glucose, and antidiabetic medication.^5^ Proportional hazards assumptions were tested and met in all Cox model analyses. While evaluating associations with all-cause mortality, model 2 also adjusted for CVD and cancer occurring before the second timepoint.

Results of the Cox regression models show mean HRs with 95% CIs. A *P* value less than .05 was considered statistically significant. Statistical analyses were performed using SAS version 9.4 (SAS Institute Inc., Cary, NC, USA).

## RESULTS

### Participant Characteristics

We included 9253 participants (51.2% women) with a mean age of 52.4±12.1 years. Approximately 29.1% of the participants smoked, 34.7% had hypertension, 5.1% had diabetes, 5.6% had a history of CVD, and 4.2% had a history of cancer (Table 1). Cohort-specific characteristics are provided in Supplemental Table 1 (available online at http://www.mayoclinicproceedings.org).

Median CRP at baseline (first visit) was 1.43 (0.63-3.38) mg/L, and during the subsequent visit was 1.59 (0.74-3.71) mg/L. While examining changes in CRP categories over time, we observed that 46.8% of participants (n=4332) had low CRP levels (ie, less than 2 mg/L) at both visits, 13.5% of participants (n=1248) changed from low (baseline) to high (second examination), 10.3% (n=955) from high to low, and 29.4% (n=2718) had high CRP levels at both visits. Participant characteristics according to longitudinal changes in CRP categories are provided in Supplemental Table 2 (available online at http://www.mayoclinicproceedings.org).

### Clinical Correlates of Longitudinal Change in CRP

In linear regression models adjusting for age, sex, and baseline CRP levels, we observed that besides older mean age and female sex, modifiable risk factors such as smoking, BMI, total cholesterol, and systolic BP were significantly associated with greater increases in CRP concentrations over time (*P*_all_<.001). In a stepwise selection model, older age, female sex, smoking, BMI, and total cholesterol remained significantly associated with change in CRP levels, with the strongest associations seen with BMI and smoking. Specifically, 1 SD higher BMI and current smoking at baseline were both associated with an approximately 15% increase in ln-standardized CRP levels (Sβ 0.151, *P*<.001 for BMI; and Sβ 0.147, *P*<.001 for smoking) (Table 2).

### Longitudinal Changes in CRP and Future Outcomes

#### Cardiovascular Disease.

During a mean follow-up of 7.8±2.0 years after the second examination, 714 cardiovascular events were observed, which corresponded to an IR of 9.9 per 1000 person-years in the total population. Individuals with persistently low CRP levels had the lowest risk of developing CVD (IR, 6.6 per 1000 person-years), those that changed categories had an intermediate risk (IR, 11.8 and 11.1 per 1000 person-years in low-high and high-low categories respectively), and those with persistently high CRP levels displayed the highest risk (IR, 16.8 per 1000 person years) (Figure).

In Cox regression models adjusting for age and sex, both baseline CRP and longitudinal changes in CRP were significantly associated with a 53% and 24% increased risk of developing CVD respectively (baseline HR per SD [HR_CRP_], 1.53; 95% CI, 1.40 to 1.67 and longitudinal changes HR per SD [HR_ΔCRP_], 1.24; 95% CI, 1.14 to 1.34) (Table 3). Effect sizes were attenuated, but trends were similar in multivariable models (HR_CRP_, 1.29; 95% CI, 1.17 to 1.43 and HR_ΔCRP_, 1.19; 95% CI, 1.09 to 1.29) (Table 3). There were no significant age*ΔCRP and sex*ΔCRP interactions (*P*>.10) (ie, age and sex did not significantly modify associations of ΔCRP with incident CVD). Cohort-specific coefficients are provided in Supplemental Table 3 (available online at http://www.mayoclinicproceedings.org).

#### Cancer.

During a mean follow-up of 13.2±3.5 years after the second examination, 1219 cancer events were recorded in the total population, which corresponded to an IR of 10.0 per 1000 person-years. Individuals with persistently low CRP levels had the lowest risk of developing cancer (IR, 7.7 per 1000 person-years), those that changed categories had an intermediate risk (IR, 12.2 and 11.9 per 1000 person-years in low-high and high-low categories, respectively), and those with persistently high CRP levels displayed the highest risk (IR, 14.0 per 1000 person-years) (Figure). In multivariable Cox-regression models, both baseline CRP and longitudinal changes in CRP were significantly associated with a 17% and 8% increased risk of developing cancer respectively (HR_CRP_, 1.17; 95% CI, 1.09 to 1.26 and HR_ΔCRP_, 1.08; 95% CI, 1.01 to 1.15) (Table 3). There were no significant age*ΔCRP and sex*ΔCRP interactions (*P*>.10). Cohort-specific coefficients are provided in Supplemental Table 3 (available online at http://www.mayoclinicproceedings.org).

Associations of baseline CRP and longitudinal changes in CRP with incident cancer after censoring the follow-up at 9 years are provided in Supplemental Table 4 (available online at http://www.mayoclinicproceedings.org).

#### All-cause Mortality.

During a mean follow-up of 12.8±3.5 years, 1656 deaths were recorded in the total population, which corresponded to a mortality rate of 14.1 per 1000 person-years. Individuals with persistently low CRP levels had the lowest risk of dying from any cause (IR, 8.8 per 1000 person-years), those that changed categories had an intermediate risk (IR, 15.8 and 15.6 per 1000 person-years in low-high and high-low categories, respectively), and those with persistently high CRP levels displayed the highest risk (IR, 21.3 per 1000 person-years in high-high category) (Figure). In multivariable Cox-regression models, both baseline CRP and longitudinal changes in CRP were significantly associated with a 29% and 10% increased risk of dying from any cause respectively (HR_CRP_, 1.29; 95% CI, 1.21 to 1.37 and HR_ΔCRP_, 1.10; 95% CI, 1.05 to 1.16) (Table 3). There were no significant age*ΔCRP and sex*ΔCRP interactions (*P*>.10). Cohort-specific coefficients are provided in Supplemental Table 3 (available online at http://www.mayoclinicproceedings.org). Associations of baseline CRP and longitudinal changes in CRP with all-cause mortality after censoring the follow-up at 9 years are provided in Supplemental Table 4 (available online at http://www.mayoclinicproceedings.org).

## DISCUSSION

In the current study, we examined associations of longitudinal changes in CRP with clinical correlates as well as with incident CVD, incident cancer, and all-cause mortality in 9253 individuals from two longitudinal community-based cohorts. We found the following: (1) higher mean age, female sex, smoking, BMI, and total cholesterol were associated with greater increases in CRP levels over time, with the strongest associations observed with higher BMI and smoking; (2) participants with CRP levels 2 mg/L or higher at both visits had the highest absolute risk of developing adverse clinical outcomes, whereas participants with CRP levels less than 2 mg/L at both visits had the lowest absolute risk; and (3) longitudinal changes in CRP levels provide additional information beyond a single baseline measurement with regard to the risk of CVD, cancer, and mortality. Taken together, these data highlight the potential value of additional CRP measurements in cardiovascular and cancer risk assessment.

### Clinical Correlates of Longitudinal Change in CRP

It has been previously observed among apparently healthy participants from the Framingham Offspring and Omni multiethnic Cohort that higher age, cigarette smoking, and BMI positively correlated with a longitudinal increase in CRP.^34^ We now show that besides the above-mentioned covariates, female sex was also strongly associated with greater increases in CRP levels over time. Similar findings were also recently reported among patients with acute coronary syndrome,^35^ highlighting the need to explore whether women would derive more benefit from targeted CRP lowering therapies than men. These observations also suggest that reducing the burden of modifiable risk factors, such as smoking and obesity, may help reduce CRP levels in the population. Interestingly, statin treatment at baseline was not associated with decrease in CRP over time. This could be because the majority of participants using statins at baseline might have already achieved lower CRP levels, and these values did not substantially decline during the 3 to 4 years of follow-up.

### Longitudinal Changes in CRP and Future Outcomes

It is well known that baseline CRP levels are strongly associated with incident CVD, cancer, and all-cause mortality in the general population.^17-20^ However, only a few studies have examined associations of serial CRP measurements with incident CVD and all-cause mortality.^21,22^ Studies examining associations of longitudinal change in CRP levels with cancer are also scarce and, to date, only one small study examined associations of serial CRP with ovarian cancer.^23^ Using data from more than 9000 individuals from two large, well-characterized cohorts, we now report that longitudinal changes in CRP may have an added value beyond baseline measurements not only for assessing cardiovascular and mortality risk, but also for assessing cancer risk. Nevertheless, in this context, change in CRP over time was robustly associated with incident cancer only in the PREVEND cohort Supplemental Table 3 and 4 (available online at http://www.mayoclinicproceedings.org). Additional studies are therefore needed to confirm the usefulness of serial CRP measurements over a single measurement for cancer risk prediction in the general population.

A noteworthy observation was the comparable absolute risk of disease in individuals belonging to low-high and high-low categories. In individuals belonging to the low-high category, average CRP levels were 1.2 mg/dL at visit 1 and 3.2 mg/dL at visit 2. Based on absolute risk of developing disease in this category, it is evident that despite low CRP levels at baseline, indicating a lower risk of developing disease, increase in CRP levels over a period of 2 to 3 years would place such individuals in an intermediate risk category, and may warrant another CRP measurement (in a few years) to better characterize disease risk. Conversely, in individuals belonging to the high-low category, average CRP levels were 3.3 mg/dL at visit 1 and 1.2 mg/dL at visit 2. Despite high CRP levels at baseline, indicating a higher risk of developing disease, a decline in CRP levels over a period of 3 years may also place such individuals in an intermediate risk category. A repeat measurement of CRP in another 2 to 3 years, if low, could potentially place these individuals at a lower risk category. The exact reason for the reduction of CRP levels in these individuals is not clear from our study, although possible explanations could include smoking cessation, reduction in body weight or initiation of statin therapy between baseline and follow-up visits.

A chronic low-grade systemic inflammatory state could contribute to CVD and cancer development through multiple mechanisms. For instance, inflammation has been shown to accelerate the ageing process and facilitate clonal expansion of cells in normal tissues, including blood cells.^36^ Indeed, data from recent studies highlight that clonal hematopoiesis of indeterminate potential is not only a risk factor for malignancies,^37,38^ but is also a risk factor for CVD,^39^ including heart failure.^40,41^

It is being increasingly recognized that directly targeting specific inflammatory processes may prevent or prolong the development of both CVD and cancer. Data from the CANTOS trial lend some support to this line of thinking as it was observed that anti-inflammatory therapy among individuals with elevated CRP and prevalent CVD resulted in reduced risk of cardiovascular as well as lung cancer events.^15,16^ Our current study highlights that baseline CRP levels as well as longitudinal increases in CRP over time in community-dwelling adults are associated with increased cardiovascular and cancer risk. Whether serial CRP measurements may help identify high-risk individuals from the general population and whether specific anti-inflammatory therapy in such individuals would also reduce the development of cardiovascular and cancer events remain to be studied.

### Study Limitations

First, because of the observational nature of our study, residual confounding cannot be excluded and causal relations cannot be determined. Second, although PREVEND and FHS participants were from two different geographical regions, the study sample was predominantly White, limiting the generalizability of our findings to other races/ethnicities. Third, samples were also largely limited to middle-to-high socioeconomic status in high-income countries, and generalizability to lower-income individuals or regions remains uncertain. Fourth, as data on exercise training and diet were not available in both cohorts, we did not include these variables in the current study. Fifth, despite pooling individual-level data from two large cohorts, we did not have adequate statistical power to examine the value of serial CRP measurements in predicting individual cancer subtypes (eg, lung and colorectal cancer).^32^ Sixth, we also acknowledge that excluding individuals with prevalent and interim cardiovascular and cancer events could have biased our sample toward healthier participants.

## CONCLUSION

In the current study, we report that initial as well as subsequent increases in CRP levels are associated with a greater risk of CVD, cancer, and death from any cause in the general population. These data suggest that periodical CRP measurements may be useful in identifying individuals at a higher risk of morbidity and mortality.

## Supplementary Material

Supplementary Material

## Figures and Tables

**FIGURE.**
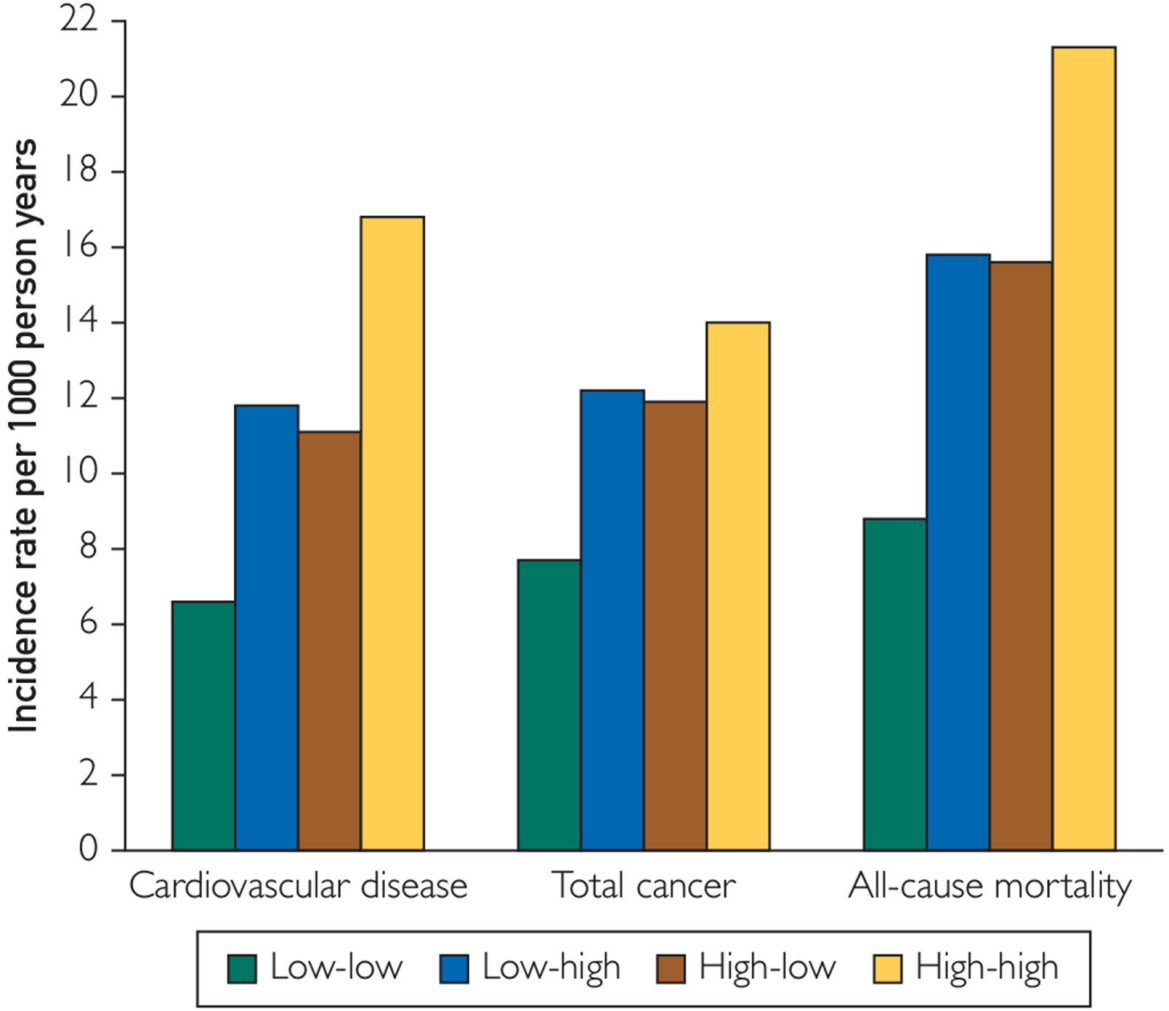
Incidence rates of cardiovascular disease, cancer, and all-cause mortality according to longitudinal changes in C-reactive protein categories. For categorizing C-reactive protein (CRP) levels, a cutpoint of 2 mg/L was used. Low-low indicates CRP levels below 2 mg/L at both visits; low-high indicates CRP levels below 2 mg/L at visit 1 and 2 mg/L or higher at visit 2; high-low indicates CRP levels of 2 mg/L or higher at visit 1 and below 2 mg/L at visit 2; and high-high indicates CRP levels of 2 mg/L or higher at both visits.

**TABLE 1.** Baseline Characteristics

Clinical characteristics	Total (n=9253)
C-reactive protein, mg/L	1.43 (0.63-3.38)
Age, y	52.4 (12.1)
Female	4733 (51.2)
Smoking	2692 (29.1)
Body mass index, kg/m^2^	26.6 (4.5)
Total cholesterol, mmol/L	5.4 (4.8-6.2)
Lipid-lowering medication	759 (8.2)
Systolic blood pressure, mm Hg	128.1 (19.2)
Antihypertensive medication	1724 (18.6)
Hypertension	3207 (34.7)
Glucose, mmol/L	4.9 (4.5-5.4)
Antidiabetic medication	234 (2.5)
Diabetes mellitus	474 (5.1)
Prevalent cardiovascular disease	515 (5.6)
Prevalent cancer	384 (4.2)

Continuous variables are as presented as mean (SD) or as median (IQR), and categorical variables as n (%). To convert total cholesterol values from mmol/L to mg/dL, multiply the value in mmol/L by 38.67. To convert glucose values from mmol/L to mg/dL, multiply the value in mmol/L by 18.018.

**TABLE 2.** Correlates of Longitudinal Change in C-reactive Protein Levels

	Age, sex, baseline CRP adjusted		Multivariable adjusted	
	Sβ	*P*	Sβ	*P*
Age, y	0.107 (0.010)	<.001	0.093 (0.010)	<.001
Female sex	0.053 (0.019)	.004	0.079 (0.019)	<.001
Smoking	0.110 (0.021)	<.001	0.147 (0.021)	<.001
Body-mass index, kg/m^2^	0.145 (0.010)	<.001	0.151 (0.010)	<.001
Total cholesterol, mmol/L	0.062 (0.010)	<.001	0.047 (0.010)	<.001
Antilipid medication	−0.022 (0.035)	.53	—	—
Glucose, mmol/L	−0.001 (0.011)	.95	—	—
Antidiabetic medication	−0.079 (0.060)	.19	—	—
Systolic blood pressure, mm Hg	0.046 (0.011)	<.001	—	—
Antihypertensive medication	0.062 (0.026)	.02	—	—
Prevalent cancer	0.008 (0.047)	.86	—	—
Prevalent cardiovascular disease	0.028 (0.041)	.50	—	—

Multivariable model included age, sex, and baseline C-reactive protein (CRP) levels along with variables with *P*<.1 in the age, sex, and baseline CRP-adjusted model. Stepwise selection was then performed, yielding the final model where all variables had a *P*<.05. Results are displayed as standardized β coefficient (Sβ), which represents the SD change in dependent variable for 1 SD change in the independent (continuous) variable or for 1 unit change in the independent (categorical) variable.

**TABLE 3.** Associations of Baseline CRP and Longitudinal Changes in CRP With Outcome

	Model 1		Model 2	
	HR (95% CI)	*P*	HR (95% CI)	*P*
Incident CVD				
Baseline CRP	1.53 (1.40-1.67)	<.001	1.29 (1.17-1.43)	<.001
ΔCRP	1.24 (1.14-1.34)	<.001	1.19 (1.09-1.29)	<.001
Incident cancer				
Baseline CRP	1.22 (1.14-1.31)	<.001	1.17 (1.09-1.26)	<.001
ΔCRP	1.10 (1.03-1.17)	.004	1.08 (1.01-1.15)	.02
All-cause mortality				
Baseline CRP	1.42 (1.34-1.50)	<.001	1.29 (1.21-1.37)	<.001
ΔCRP	1.13 (1.07-1.19)	<.001	1.10 (1.05-1.16)	<.001

Baseline C-reactive protein (CRP) concentrations were natural-log (ln) transformed. Longitudinal change in CRP was calculated as the difference in ln-CRP concentrations between the two consecutive exams (ie, ΔCRP equals ln-transformed CRP_2_ minus ln-transformed CRP_1_). Baseline CRP and ΔCRP were then standardized and simultaneously entered in all models. Model 1 adjusted for age at baseline and sex. Model 2 additionally adjusted for the following baseline covariates: smoking, body mass index, total cholesterol, lipid-lowering medication, glucose, antidiabetic medication, systolic blood pressure and antihypertensive medication. In cardiovascular disease (CVD) models, individuals with prevalent and interim CVD were excluded. In cancer models, individuals with prevalent and interim cancer were excluded. While examining associations with all-cause mortality, model 2 was additionally adjusted for CVD and cancer events before the second visit.

## Abbreviations and Acronyms:

BMI: body mass index
CRP: C-reactive protein
CVD: cardiovascular disease
