# Supplementary material for: Association of Initial and Longitudinal Changes in C-reactive Protein With the Risk of Cardiovascular Disease, Cancer, and Mortality
Source: Mayo Clin Proc. Author manuscript; Available in PMC 2023 Dec 6. (PMC10698556; doi:10.1016/j.mayocp.2022.10.013)
Supplement: Supplementary Material [file NIHMS1946558-supplement-Supplementary_Material.pdf]

Supplemental Table 1. Baseline characteristics stratified by cohorts

Clinical characteristics	PREVEND (n=6258)	FHS (n=2995)
C-reactive protein, mg/L	1.21 (0.54-2.83)	2.00 (0.93-4.61)
Age, years	50 (12)	58 (10)
Female, n (%)	3139 (50.2)	1594 (53.2)
Smoking, n (%)	2251 (36.0)	441 (14.7)
Body mass index, kg/m^2	26.0 (4.1)	27.9 (5.1)
Cholesterol, mmol/L	5.5 (4.8-6.3)	5.2 (4.7-5.9)
Lipid-lowering medication, n (%)	389 (6.2)	370 (12.4)
Systolic blood pressure, mm Hg	128.2 (19.6)	127.8 (18.3)
Antihypertensive medication, n (%)	905 (14.5)	819 (27.4)
Hypertension, n (%)	1997 (31.9)	1210 (40.4)
Glucose, mmol/L	4.7 (4.3-5.1)	5.4 (5.1-5.9)
Antidiabetic medication, n (%)	91(1.5)	143 (4.8)
Diabetes mellitus, n (%)	201 (3.2)	273 (9.1)
Prevalent cardiovascular disease, n (%)	363 (5.8)	152 (5.1)
Prevalent cancer, n (%)	182 (2.9)	202 (6.7)

Continuous variables are as presented as mean (SD) or as median (P25-P75), and categorical variables as n (%).

Supplemental Table 2. Participant characteristics according to longitudinal changes in C-reactive protein (CRP) categories

**CLINICAL CHARACTERISTICS**	**Low-low**	**Low-high**	**High-low**	**High-high**
Participants, n (%)	4332	1248	955	2718
CRP @ visit 1, mg/L	0.64 (0.36-1.06)	1.22 (0.82-1.59)	3.30 (2.48-5.31)	4.62 (3.06-7.93)
CRP @ visit 2, mg/L	0.76 (0.43-1.20)	3.17 (2.49-4.81)	1.19 (0.78-1.61)	4.94 (3.25-8.02)
Age, years	49.9 (11.8)	53.1 (11.9)	52.5 (12.5)	56.2 (11.3)
Female, n (%)	2101 (48.5)	617 (49.4)	462 (48.4)	1553 (57.1)
Smoking, n (%)	1141 (26.3)	374 (30.0)	306 (32.0)	871 (32.1)
Body mass index, kg/m^2	25.1 (3.5)	26.5 (3.8)	26.7 (4.1)	30.1 (5.7)
Cholesterol, mmol/L	5.3 (4.7-6.1)	5.5 (4.8-6.3)	5.3 (4.7-6.1)	5.6 (5.0-6.4)
Lipid-lowering medication, n (%)	284 (6.6)	107 (8.6)	88 (9.2)	280 (10.3)
Systolic blood pressure, mm Hg	124.6 (17.8)	128.2 (19.3)	129.2 (19.5)	133.1 (20.0)
Antihypertensive medication, n (%)	506 (11.7)	249 (20.0)	207 (21.7)	762 (28.0)
Hypertension, n (%)	1103 (25.5)	448 (35.9)	358 (37.5)	1298 (47.8)
Glucose, mmol/L	4.8 (4.4-5.2)	4.9 (4.5-5.4)	5.0 (4.5-5.5)	5.2 (4.7-5.8)
Antidiabetic medication, n (%)	60 (1.4)	25 (2.0)	37 (3.9)	112 (4.1)
Diabetes mellitus, n (%)	116 (2.7)	39 (3.1)	70 (7.4)	249 (9.2)
Prevalent cancer, n (%)	140 (3.2)	54 (4.3)	40 (4.2)	150 (5.5)
Interim cancer, n (%)	57 (1.3)	30 (2.4)	16 (1.7)	57 (2.1)
Prevalent cardiovascular disease, n (%)	187 (4.3)	77 (6.2)	57 (6.0)	194 (7.1)
Interim cardiovascular disease, n (%)	76 (1.8)	34 (2.7)	27 (2.8)	85 (3.1)

Continuous variables are as presented as mean (SD) or as median (P25-P75), and categorical variables as n (%). For categorizing CRP levels, a cutpoint of 2 mg/L was used; low-low indicates CRP levels less than 2mg/L at both visits; low-high indicates CRP levels less than 2mg/L at visit 1 and 2mg/L or higher at visit 2; high-low indicates CRP levels 2mg/L or higher at visit 1 and less than 2mg/L at visit 2; and high-high indicates CRP levels 2mg/L or higher at both visits.

Supplemental Table 3. Cohort-specific associations of baseline CRP and longitudinal changes in CRP with outcome

	PREVEND (n=6258)		FHS (n=2995)	
	HR (95%CI)	*P*-value	HR (95%CI)	*P*-value
**Incident CVD**				
Baseline CRP	1.32 (1.19-1.46)	<.001	1.34 (1.16-1.55)	<.001
ΔCRP	1.13 (1.04-1.23)	.005	1.18 (1.06-1.32)	.003
**Incident Cancer**				
Baseline CRP	1.22 (1.10-1.34)	<.001	1.12 (1.01-1.23)	.03
ΔCRP	1.18 (1.09-1.28)	<.001	1.03 (0.95-1.12)	.44
**All-cause Mortality**				
Baseline CRP	1.23 (1.13-1.35)	<.001	1.33 (1.21-1.46)	<.001
ΔCRP	1.10 (1.02-1.18)	.01	1.10 (1.02-1.19)	.01

Baseline C-reactive protein (CRP) concentrations were natural-log (ln) transformed. Longitudinal change in CRP (ΔCRP) was calculated as the difference in ln-CRP concentrations between the two consecutive exams. Baseline CRP and ΔCRP were then standardized and simultaneously entered in all models. HR represents hazard ratio and CI represents confidence interval. All models were adjusted for age, sex, smoking, body mass index, total cholesterol, anti-lipid medication, glucose, antidiabetic medication, systolic blood pressure, antihypertensive medication. In cardiovascular disease (CVD) models, individuals with prevalent and interim CVD were excluded. In cancer models, individuals with prevalent and interim cancer were excluded. While examining associations with all-cause mortality, models were additionally adjusted for CVD and cancer events before the second visit.

Supplemental Table 4. Associations of baseline and longitudinal changes in CRP with incident cancer and mortality: follow-up censored at 9 years

	**TOTAL (n=9253)**		**PREVEND (n=6258)**		**FHS (n=2995)**	
	HR (95% CI)	*P*-value	HR (95% CI)	*P*-value	HR (95% CI)	*P*-value
**Incident Cancer**						
Baseline CRP	1.25 (1.14-1.38)	<.001	1.43 (1.21-1.70)	<.001	1.19 (1.06-1.35)	.005
ΔCRP	1.16 (1.07-1.26)	.001	1.30 (1.13-1.51)	<.001	1.10 (0.99-1.22)	.08
**All-cause Mortality**						
Baseline CRP	1.32 (1.21-1.45)	<.001	1.23 (1.09-1.40)	.001	1.42 (1.24-1.63)	<.001
ΔCRP	1.18 (1.09-1.27)	<.001	1.17 (1.05-1.29)	.003	1.18 (1.06-1.32)	.003

The mean follow-up duration for incident cancer and all-cause mortality in the total population were 8.8±0.75 years and 8.4±1.67 years respectively. Baseline C-reactive protein (CRP) concentrations were natural-log (ln) transformed. Longitudinal change in CRP (ΔCRP) was calculated as the difference in ln-CRP concentrations between the two consecutive exams. Baseline CRP and ΔCRP were then standardized and simultaneously entered in all models. HR represents hazard ratio and CI represents confidence interval. Models were adjusted for age, sex, smoking, body mass index, total cholesterol, anti-lipid medication, glucose, antidiabetic medication, systolic blood pressure, antihypertensive medication. In cancer models, individuals with prevalent and interim cancer were excluded. While examining associations with all-cause mortality, models were additionally adjusted for CVD and cancer events before the second visit.
